# Supplementary material for: Nanopore sequencing at Mars, Europa, and microgravity conditions
Source: NPJ Microgravity. 2020 Sep 7;6:24. doi: 10.1038/s41526-020-00113-9 (PMC7477557; doi:10.1038/s41526-020-00113-9)
Supplement: Supplementary file 1 — Supplementary Information [file 41526_2020_113_MOESM1_ESM.pdf]

Supplementary Information: Nanopore Sequencing at Mars, Europa and Microgravity Conditions

Christopher E. Carr[1,2,*], Noelle C. Bryan[1], Kendall N. Saboda[1], Srinivasa A. Bhattaru[3], Gary Ruvkun[2], Maria T. Zuber[1]

[1]Massachusetts Institute of Technology, Department of Earth, Atmospheric and Planetary Sciences, Cambridge, MA, USA. [2]Massachusetts General Hospital, Department of Molecular Biology, Boston, MA, USA.[3]Massachusetts Institute of Technology, Department of Aeronautics and Astronautics, Cambridge, MA, USA

*Correspondence: 77 Massachusetts Ave Room 54-418, Cambridge MA 02138, USA. chrisc@mit.edu, +1-617-253-0786.

Here we provide supplementary figures (11) and tables (9).

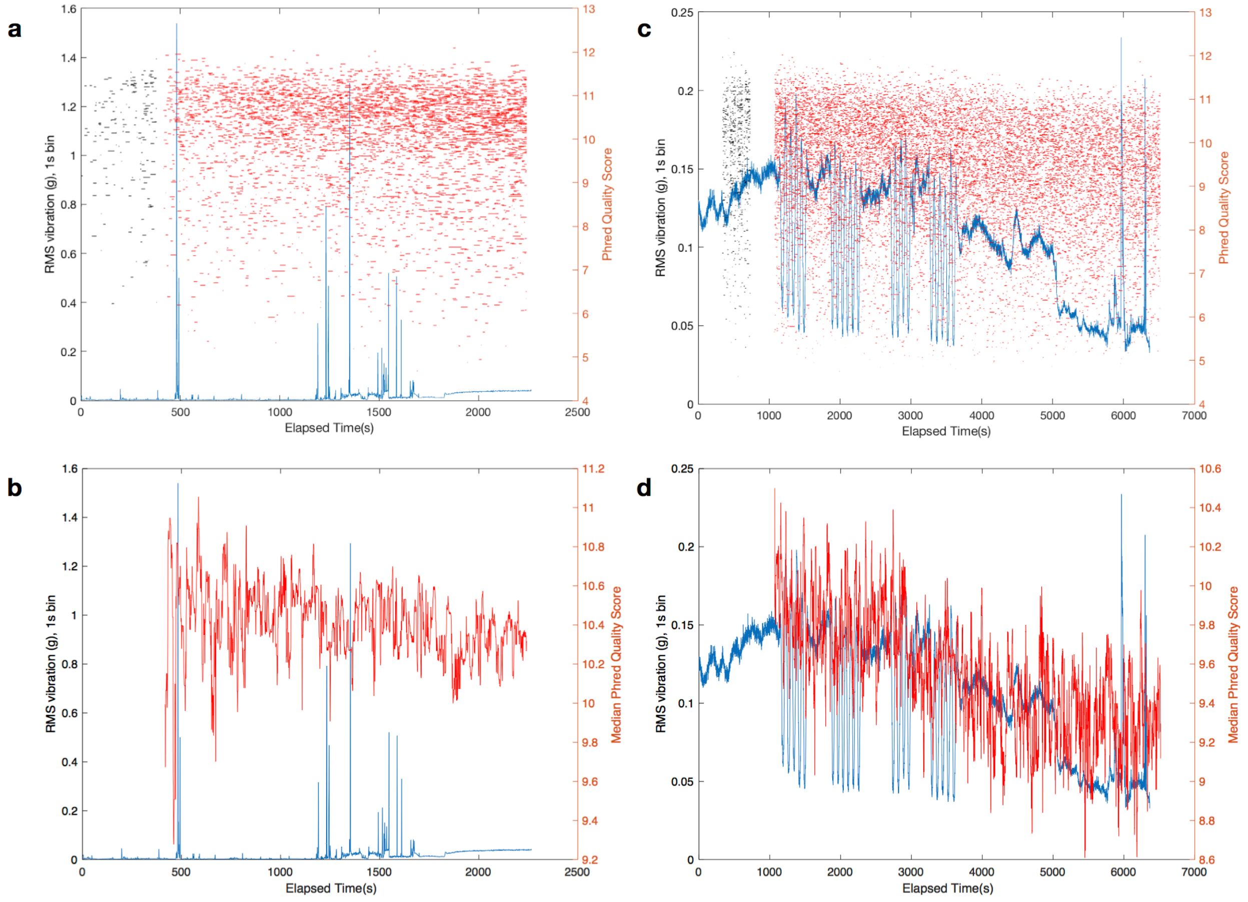

Supplementary Fig. 1. RMS vibration and Sequence Read Quality. a Ground RMS vibration (blue) and read quality (mux=grey, run=red). **b** Ground RMS vibration (blue) and median read quality (red). **c** Flight RMS vibration (blue) and read quality (mux=greay, run=red). **d** Flight RMS vibration (blue) and median read quality (red). In panels **a-b**, each horizontal line represents one sequencing read. Mux reads are excluded from panels **b**, **d**.

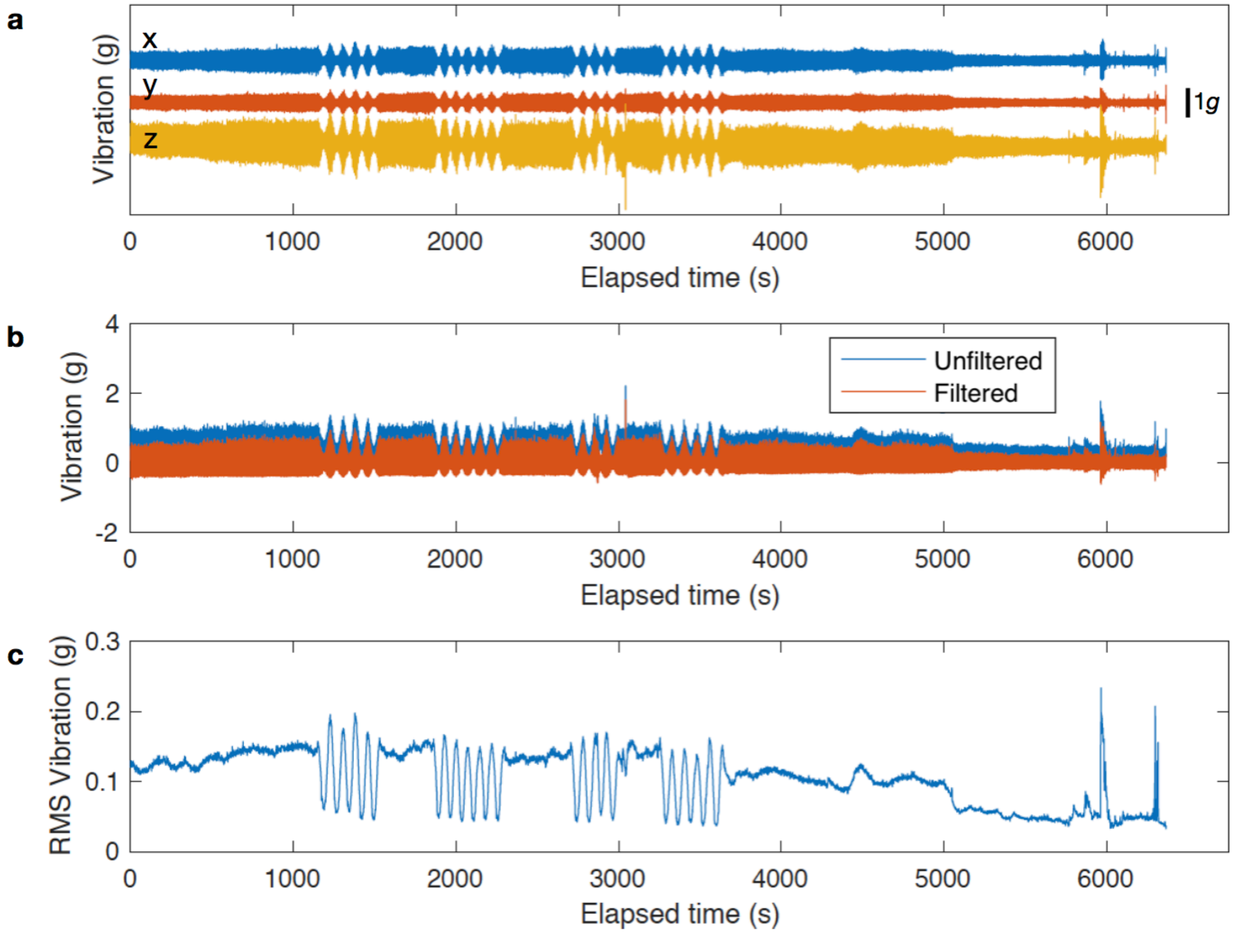

Supplementary Fig. 2. Vibration during flight (overview). a Unfiltered vibration measurements after mean removal (1.5 g offsets for display only: x +3 g, y + 1.5 g, z +0 g). Scale bar: 1g. **b** *g*-level equivalent vibration pre- and post-filtering. **c** Root-mean-square (RMS) vibration profile (1 second bin).

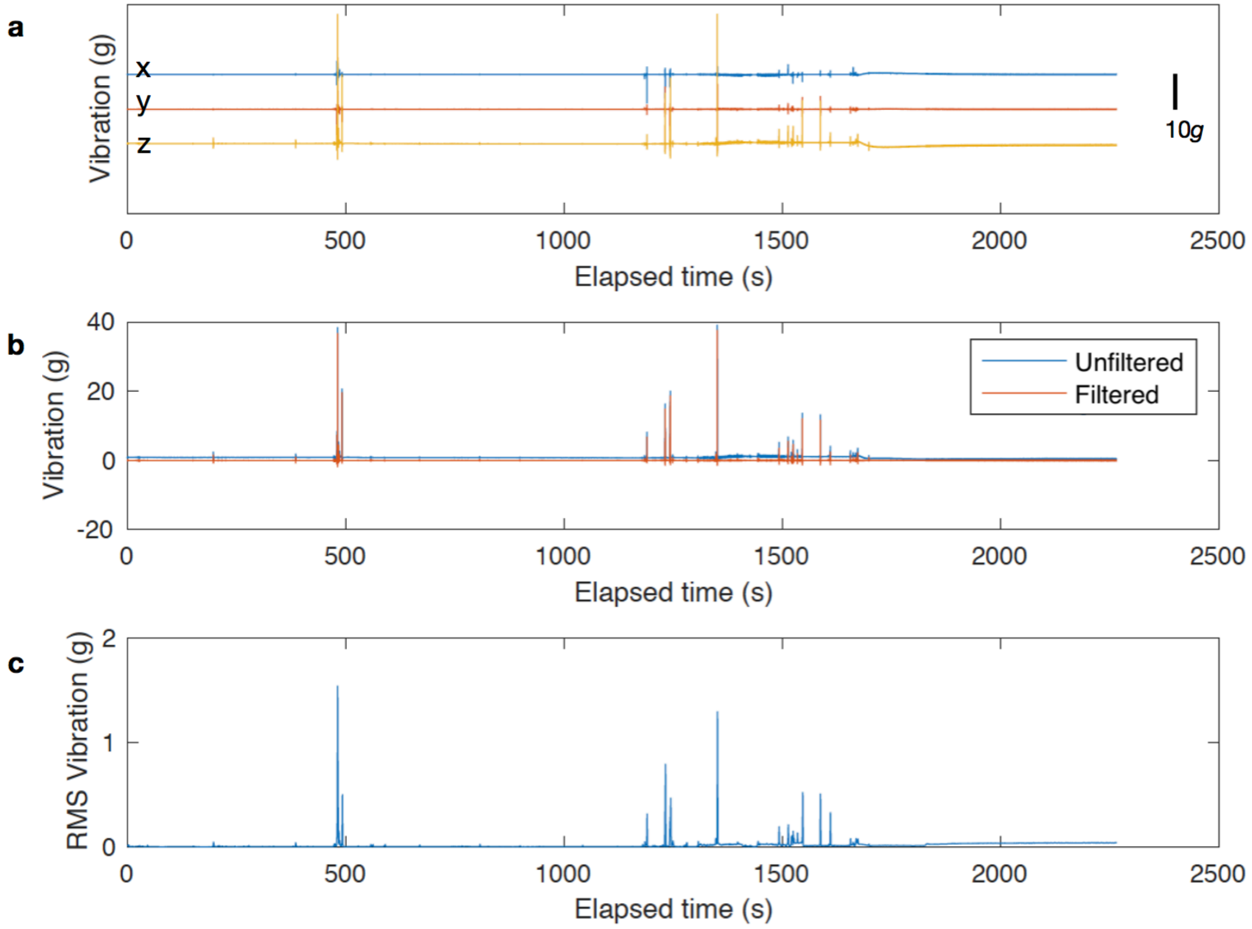

Supplementary Fig. 3. Vibration during ground operations (overview). a Unfiltered vibration measurements after mean removal (10g offsets for display). Scale bar: 10g. **b** *g*-level equivalent vibration pre- and post-filtering. **c** Root-mean-square (RMS) vibration profile (1 second bin).

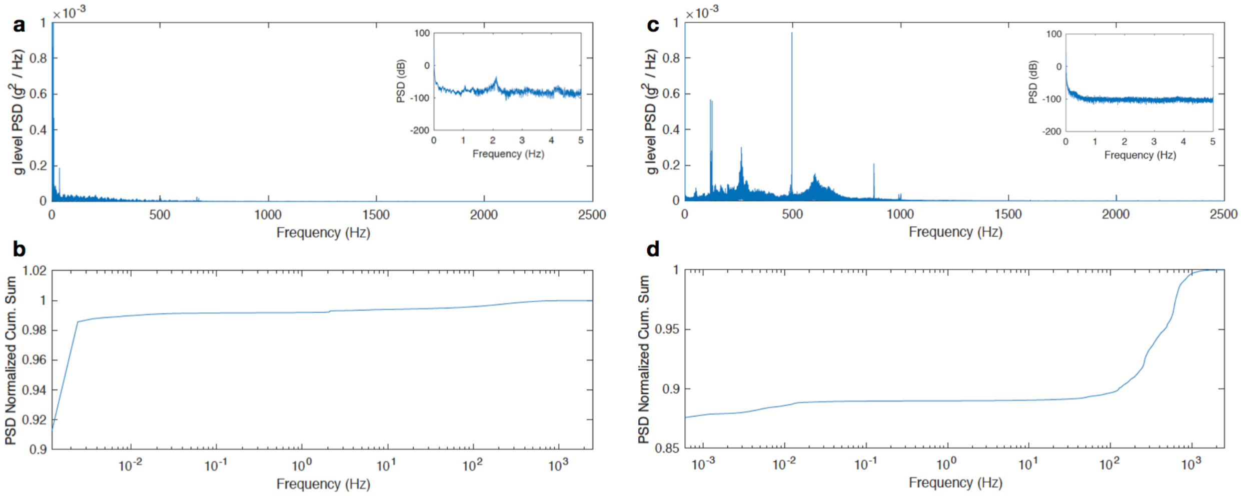

Supplementary Fig. 4. Power spectral density (PSD) of vibration *g*-level equivalent. a Ground PSD. **b** Cumulative sum of Ground PSD. **c** Flight PSD. **d** Cumulative sum of Flight PSD.

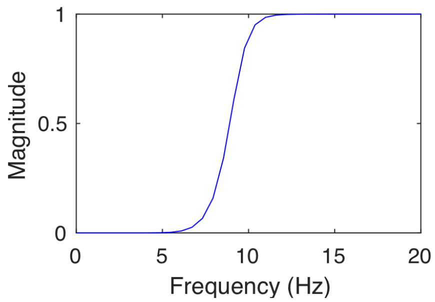

Supplementary Fig. 5. Frequency response of vibration filter. Phase is not shown as it is not relevant due to use of zero-phase filtering.

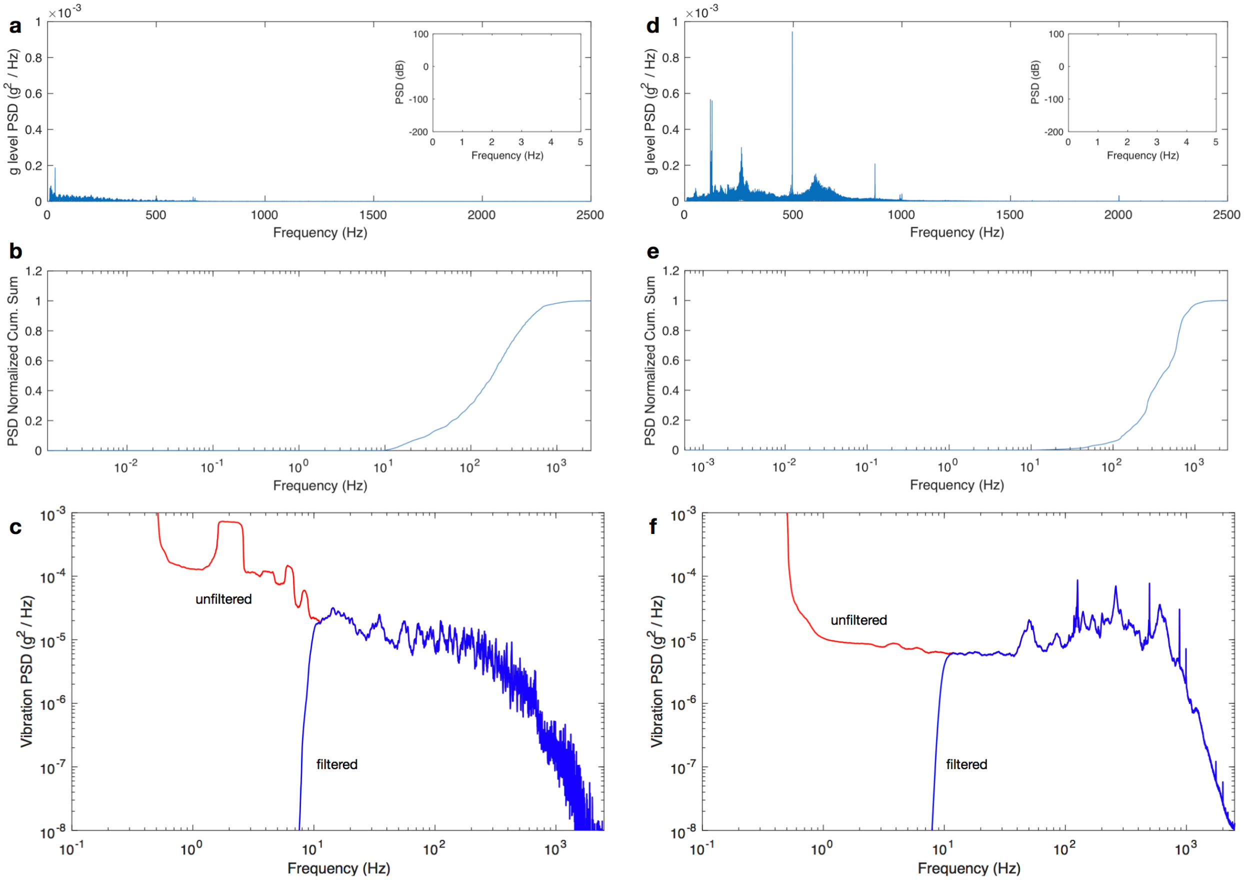

Supplementary Fig. 6. Power spectral density (PSD) of vibration *g*-level equivalent after high-pass filtering. a Ground PSD. **b** Cumulative sum of Ground PSD. **c** Ground PSD smoothed with window size of 1 Hz (red=unfiltered, blue=filtered). **d** Flight PSD. **e** Cumulative sum of Flight PSD. **f** Flight PSD smoothed with window size of 1 Hz (red=unfiltered, blue=filtered).

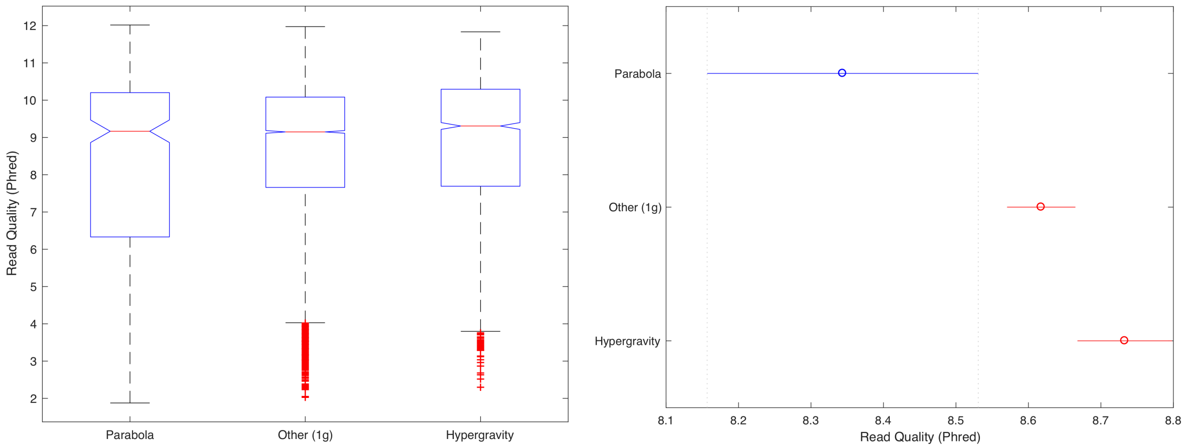

Supplementary Fig. 7. Effect of phase of flight on read quality. Distribution of read quality ($q_{\bar{p}}$) as a function of phase of flight (left) and Tukey Honestly Significant Difference (HSD) test of group means (right). The "transition" phase of flight is excluded as only 7 reads fell wholly within transition periods. Boxes (left) indicate median (red line) and 25% to 75% percentiles, with whiskers extending to approximately 2.7σ for normal data, outliers drawn as red +. Group means (right) are shown with confidence intervals (Supplementary Table 5).

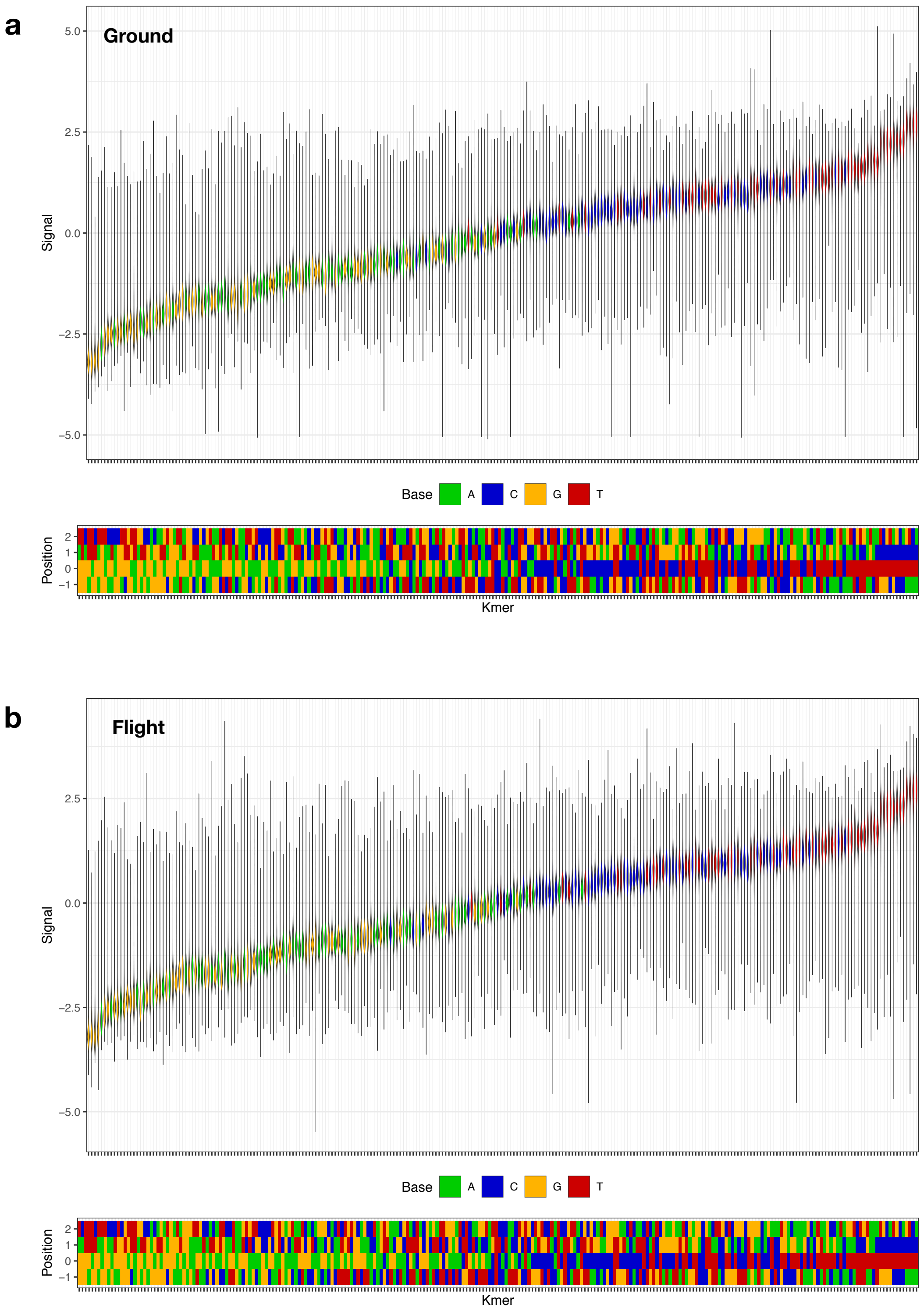

Supplementary Fig. 8. K-mer Signal Levels. Associations of ionic current with patterns of bases (k-mers) were evaluated using the *tombo plot kmer* command for the Ground (**a**) and Flight (**b**) datasets respectively. The pattern of k-mers is nearly identical, with minor changes in k-mer order; qualitatively, the signal profiles for Ground and Flight are nearly identical despite the large average difference in vibration. Signal for each k-mer is represented as a violin plot.

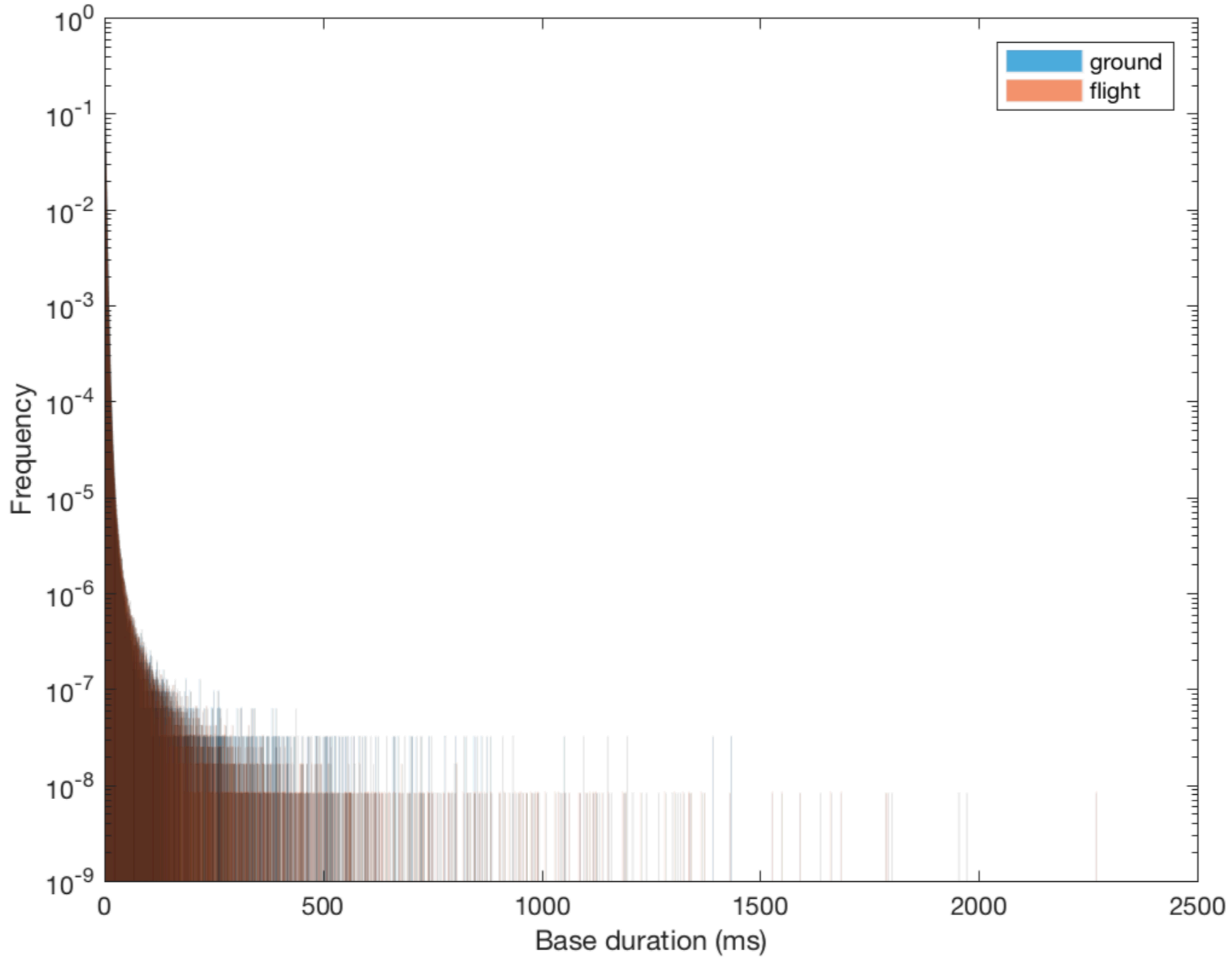

Supplementary Fig. 9. Nanopore translocation time as measured by alignment of ionic current to the genomic reference: full range. Ground (blue), flight (light brown), both (dark brown).

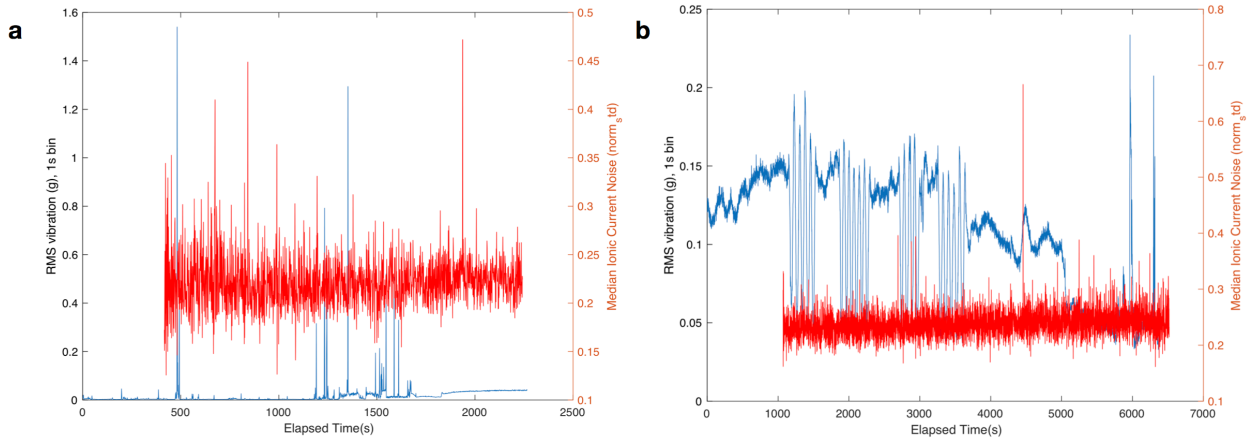

Supplementary Fig. 10. RMS vibration and Ionic Current Noise. a Ground RMS vibration (blue) and median ionic current noise (red, 1s bin). **b** Flight RMS vibration (blue) and median ionic current noise (red, 1s bin).

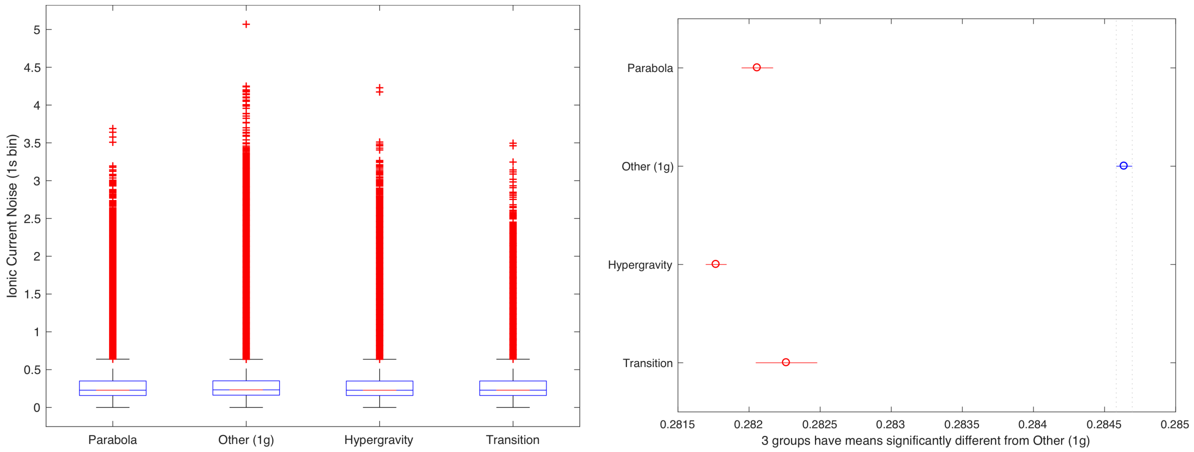

Supplementary Fig. 11. Effect of phase of flight on ionic current noise. Distribution of ionic current noise estimate for each aligned genomic base as a function of phase of flight (left) and Tukey Honestly Significant Difference (HSD) test of group means (right). Boxes (left) indicate median (red line) and 25% to 75% percentiles, with whiskers extending to approximately 2.7σ for normal data, outliers drawn as red +. Group means (right) are shown with confidence intervals (Supplementary Table 9).

Supplementary Table 1. Sequencing Statistics.

Dataset	Ground	Flight
**Mux Reads**	197	850
**Non-Mux Reads**	5,096	17,383
**Total Reads**	5,293	18,233
**Basecalled Reads**	5,257	18,188
**Basecalled Reads > Q6.5**	4,436	15,121
**Tombo-Aligned Reads**	4,459	15,570
**Basecalled Bases**	35,499,061	132,453,470
**Tombo-Aligned Bases**	31,183,016	118,786,557
**Trimmed (Adaptor) Bases**	139,482	486,894
**Non-Adaptor Bases**	35,359,579	131,966,576
**% Non-Adaptor Bases**	99.61%	99.63%
**% Aligned Bases**	87.84%	89.68%

Supplementary Table 2. Timing Data.

Sequencer	MinION - Ground	MinION - Flight
**Mux experiment start time**	2017-11-17T14:59:03Z	2017-11-17T18:34:20Z
**Run experiment start time**	2017-11-17T15:06:12Z	2017-11-17T18:46:32Z
**Max read start time in samples (s)**	7339607	21823499
**Corresponding duration (s)**	4969	4981
**Sampling rate (Hz)**	4000	4000
**Sequencing duration (s)**	1836.144	5457.12
**Mux duration (s)**	429	732
**Total sequencing duration (s)**	2265.144	6189.12
**Total sequencing duration (min)**	37.7524	103.152
**Accelerometer**	**SlamStick - Ground**	**SlamStick - Flight**
**Start time**	2017-11-17T14:59:26Z	2017-11-17T18:28:51Z
**Timing Offsets**	**MinION - Ground**	**MinION - Flight**
**Mux Offset (s)**	-23	329
**Run Offset (s)**	406	1061

Timing offsets are added to sequencing time to get accelerometer elapsed time.

Supplementary Table 3. Ground Operations: Do time and RMS vibration predict median sequence quality?

Dataset	Estimate	SE	t-stat	p-value
**Measurements**	1827			
**Error degrees of freedom**	1825			
**Regression RMS Error**	0.182			
**Adjusted R-squared**	0.0602			
**F-statistic vs. constant model**	118			1.1E-26
**Intercept**	10.525	0.01157	909.63	0
**Time (s)**	-8.7908E-5	8.0926E-6	-10.863	1.1E-26

Above table is final model from stepwise linear regression. Process from constant model:
pValue for adding Time is 1.1145e-26
pValue for adding Vibration is 0.080905
1. Adding Time, FStat = 117.9991, pValue = 1.114479e-26
pValue for adding Vibration is 0.89155
No candidate terms to remove

Supplementary Table 4. Flight Operations: Do time, RMS vibration, and *g*-level predict median sequence quality?

Dataset	Estimate	SE	t-stat	p-value
**Measurements**	2931			
**Error degrees of freedom**	2927			
**Regression RMS Error**	0.200			
**Adjusted R-squared**	0.275			
**F-statistic vs. constant model**	371			2.1E-204
**Intercept**	9.9413	0.028948	343.42	0
**Time**	-9.8603E-05	1.1189E-05	-8.8126	2.05E-18
**g-level**	0.14773	0.025729	5.7416	1.03E-08
**Time:g-level**	-4.2902E-05	9.9399E-06	-4.3161	1.64E-05

Above table is final model from stepwise linear regression. Process from constant model:
pValue for adding Time is 2.4094e-197
pValue for adding Vibration is 1.9926e-18
pValue for adding g-level is 0.00017903
1. Adding Time, FStat = 1051.3572, pValue = 2.4093671e-197
pValue for adding Vibration is 1.4909e-06
pValue for adding g-level is 1.2423e-07
2. Adding g-level, FStat = 28.0928, pValue = 1.24226e-07
pValue for adding Vibration is 0.25185
pValue for adding Time:g-level is 1.6406e-05
3. Adding Time:g-level, FStat = 18.6289, pValue = 1.6406e-05
pValue for adding Vibration is 0.4692
No candidate terms to remove

Supplementary Table 5. Flight Operations: Does read quality differ between phases of flight?

Source	SS	df	MS	F	Prob>F
**Groups**	55.6	2	27.8023	7.16	0.0008
**Error**	52548.9	13531	3.8836		
**Total**	52604.5	13533			
**Group1**	**Group 2**	**CI (low)**	**HSD**	**CI (high)**	**p-value**
**Parabola**	**Other (1g)**	-0.5081	-0.2739	-0.0397	0.0168
**Parabola**	**Hypergravity**	-0.6415	-0.3892	-0.1369	0.0009
**Other (1g)**	**Hypergravity**	-0.2277	-0.1153	-0.0028	0.0431

Group means: 8.3437 (parabola), 8.6177 (other), 8.7329 (hypergravity). HSD = Honest Significant Difference. CI is 95% percentile. This test is by definition two-sided.

Supplementary Table 6. Flight Operations: Does parabola duration predict coverage?

Dataset	Estimate	SE	t-stat	p-value
**Measurements**	20			
**Error degrees of freedom**	18			
**Regression RMS Error**	1.3			
**Adjusted R-squared**	0.807			
**F-statistic vs. constant model**	80.2			4.73E-08
**Intercept**	-6.7619	1.8938	-3.5706	2.19E-03
**Duration (s)**	0.81335	0.09081	8.9565	4.73E-08

Above table is final model from stepwise linear regression. Process from constant model:
pValue for adding Duration is 4.7332e-08
1. Adding Duration, FStat = 80.2195, pValue = 4.73317e-08
No candidate terms to add
No candidate terms to remove

Supplementary Table 7. Ground Operations: Do time and RMS vibration predict ionic current noise?

Dataset	Estimate	SE	t-stat	p-value
**Measurements**	1827			
**Error degrees of freedom**	1823			
**Regression RMS Error**	0.0267			
**Adjusted R-squared**	0.00903			
**F-statistic vs. constant model**	6.55			0.000211
**Intercept**	0.21866	0.0018111	120.74	0
**Time**	2.1177E-06	1.3858E-06	1.5281	0.12666
**Vibration**	-0.077314	0.024691	-3.1313	0.0017679
**Time:Vibration**	4.7315E-05	2.3164E-05	2.0426	0.041235

Above table is final model from stepwise linear regression. Process from constant model:
pValue for adding Time is 0.012543
pValue for adding Vibration is 0.009305
1. Adding Vibration, FStat = 6.7777, pValue = 0.009305
pValue for adding Time is 0.0033302
2. Adding Time, FStat = 8.6399, pValue = 0.0033302
pValue for adding Time:Vibration is 0.041235
3. Adding Time:Vibration, FStat = 4.1722, pValue = 0.041235
No candidate terms to add
No candidate terms to remove

Supplementary Table 8. Flight Operations: Do time, RMS vibration, and *g*-level predict ionic current noise?

Dataset	Estimate	SE	t-stat	p-value
**Measurements**	2931			
**Error degrees of freedom**	2929			
**Regression RMS Error**	0.0208			
**Adjusted R-squared**	0.0137			
**F-statistic vs. constant model**	41.8			1.18E-10
**Intercept**	0.22626	0.0012148	186.26	0
**Time (s)**	2.9385E-06	4.5455E-07	6.4647	1.18E-10

Above table is final model from stepwise linear regression. Process from constant model:
pValue for adding Time is 1.185e-10
pValue for adding Vibration is 0.99466
pValue for adding g-level is 0.28286
1. Adding Time, FStat = 41.792, pValue = 1.18499e-10
pValue for adding Vibration is 0.27136
pValue for adding g-level is 0.36804
No candidate terms to remove
Note: extending analysis time to entire flight does not change any of our conclusions.

Supplementary Table 9. Flight Operations: Does ionic current noise differ between phases of flight?

Source	SS	df	MS	F	Prob>F
**Groups**	183.6898	3	61.2299	1.6204e+03	0
**Error**	4.2091e+06	111393248	0.0378		
**Total**	4.2093e+06	111393251			
**Group1**	**Group 2**	**CI (low)**	**HSD**	**CI (high)**	**p-value**
**Parabola**	Other (1g)	-0.00275	-0.00258	-0.00241	3.77E-09
**Parabola**	Hypergravity	0.00010	0.00029	0.00048	5.96E-04
**Parabola**	Transition	-0.00052	-0.00020	0.00011	0.345
**Other (1g)**	Hypergravity	0.00275	0.00287	0.00299	3.77E-09
**Other (1g)**	Transition	0.00210	0.00238	0.00265	3.77E-09
**Hypergravity**	Transition	-0.00079	-0.00049	-0.00020	8.55E-05

Group means: 0.2818 (hypergravity), 0.2821 (parabola), 0.2823 (transition), 0.2846 (other/1g). HSD = Honest Significant Difference. CI is 95% percentile. This test is by definition two-sided.
